# The lncRNA HOXA11-AS functions as a competing endogenous RNA to regulate PADI2 expression by sponging miR-125a-5p in liver metastasis of colorectal cancer

**DOI:** 10.18632/oncotarget.19956

**Published:** 2017-08-03

**Authors:** Dong Chen, Qiang Sun, Lufei Zhang, Xiaohu Zhou, Xiaofei Cheng, Dongkai Zhou, Feng Ye, Jianjiang Lin, Weilin Wang

**Affiliations:** ^1^ Department of Colorectal Surgery, First Affiliated Hospital, Zhejiang University School of Medicine, Hangzhou, China; ^2^ Key Laboratory of Precision Diagnosis and Treatment for Hepatobiliary and Pancreatic Tumor of Zhejiang Province, Hangzhou, China; ^3^ State Key Laboratory & Collaborative Innovation Center for Diagnosis and Treatment of Infectious Disease, First Affiliated Hospital, Zhejiang University School of Medicine, Hangzhou, China; ^4^ Division of Hepatobiliary and Pancreatic Surgery, First Affiliated Hospital, Zhejiang University School of Medicine, Hangzhou, China

**Keywords:** colorectal cancer (CRC), HOXA11 antisense RNA (HOXA11-AS), liver metastasis, long noncoding RNA (lncRNA), peptidyl arginine deiminase 2 (PADI2)

## Abstract

Several long non-coding RNAs (lncRNAs) play important roles in the regulation of liver metastasis in colorectal cancer (CRC) patients. We previously described the potential involvement of HOMEOBOX A11 (HOXA11) antisense RNA (HOXA11-AS), miR-125a-5p, and peptidyl arginine deiminase 2 (PADI2) in promoting liver metastasis in CRC patients. In the present study, we verified the significant upregulation of HOXA11-AS and PADI2, as well as the downregulation of miR-125a-5p, in CRC patients with liver metastasis. Overexpression and knockdown studies of HOXA11-AS or PADI2, as well as gain-/loss-of-function studies of miR-125a-5p, revealed a positive correlation between HOXA11-AS and PADI2 and a negative correlation with miR-125a-5p in the regulation of liver metastasis in CRC cell lines. Overall, we conclude that HOXA11-AS promotes liver metastasis in CRC by functioning as a miR-125a-5p sponge and describe a novel HOXA11-AS–miR-125a-5p–PADI2 regulatory network involved in CRC liver metastasis.

## INTRODUCTION

According to the latest statistics, colorectal cancer (CRC) is the third most commonly diagnosed cancer in the United States [1] and the fifth most diagnosed cancer in China [2, 3]. Metastasis is the primary cause of death for CRC patients, and the liver is the primary site of metastatic lesions due to portal drainage [4]. Therefore, the early diagnosis and treatment of liver metastasis in CRC patients are important for improved mortality and survival. Currently, the diagnosis of liver metastasis is based on imaging; however, existing methods for the early diagnosis of liver metastasis are far from sufficient [5]. Therefore, identifying novel functional biomarkers is imperative.

Long non-coding RNAs (lncRNAs; 200–100,000 nucleotides in length) participate in the biological and pathological processes of CRC, including apoptosis, proliferation, and metastasis [4, 6, 7]. Emerging evidence indicates that lncRNAs act as competing endogenous RNAs (ceRNAs) or molecular sponges that modulate microRNAs (miRNAs) [8]. Nevertheless, most functions of lncRNAs have not been clarified.

HOMEOBOX A11 antisense RNA (HOXA11-AS) is a newly identified lncRNA. Its expression is related to glioma, uterine cervix carcinoma, epithelial ovarian cancer, and lung adenocarcinoma [9–12]. Moreover, significant down-regulation of HOXA11-AS in CRC was reported recently, although its mechanism of action was not defined [13]. However, the pattern of HOXA11-AS expression and its role in the liver metastasis of CRC have not been reported.

miR-125a-5p acts as a suppressor of hepatocellular carcinoma, gastric cancer, breast cancer, lung cancer, glioma, and melanoma, with several proven target molecules, including SIRT7, PI3K, E2F3, ERBB2, TSTA3, HDAC4, HDAC5, EGFR, Gab2, and Lin28B [14–24].

Peptidyl arginine deiminase 2 (PADI2) was first reported as an important biomarker for HER2/ERBB2^+^ breast cancers [25]. Moreover, several recent studies have suggested that PADI2 is involved in tumorigenesis and plays an important role in the progression of skin neoplasia, myeloma, breast cancer, and CRC, although its tumorigenic effect and mechanism are largely unknown [26–29]. In addition to tumorigenesis, PADI2 is believed to promote the epithelial-mesenchymal transition during the progression of skin neoplasms [26]; however, the expression of PADI2 in liver metastasis of CRC has not been reported.

In this study, HOXA11-AS expression was up-regulated in the tissues of CRC patients with liver metastasis. The knockdown and overexpression of HOXA11-AS inhibited and promoted the migration and invasion of colon cancer cells, respectively. Moreover, we confirmed that HOXA11-AS functions as a ceRNA to regulate PADI2 expression by sponging miR-125a-5p. The HOXA11-AS/miR-125a-5p/PADI2 regulatory network may represent a novel therapeutic target for liver metastasis of CRC.

## RESULTS

### HOXA11-AS expression in CRC with liver metastasis

**Figure 1:**
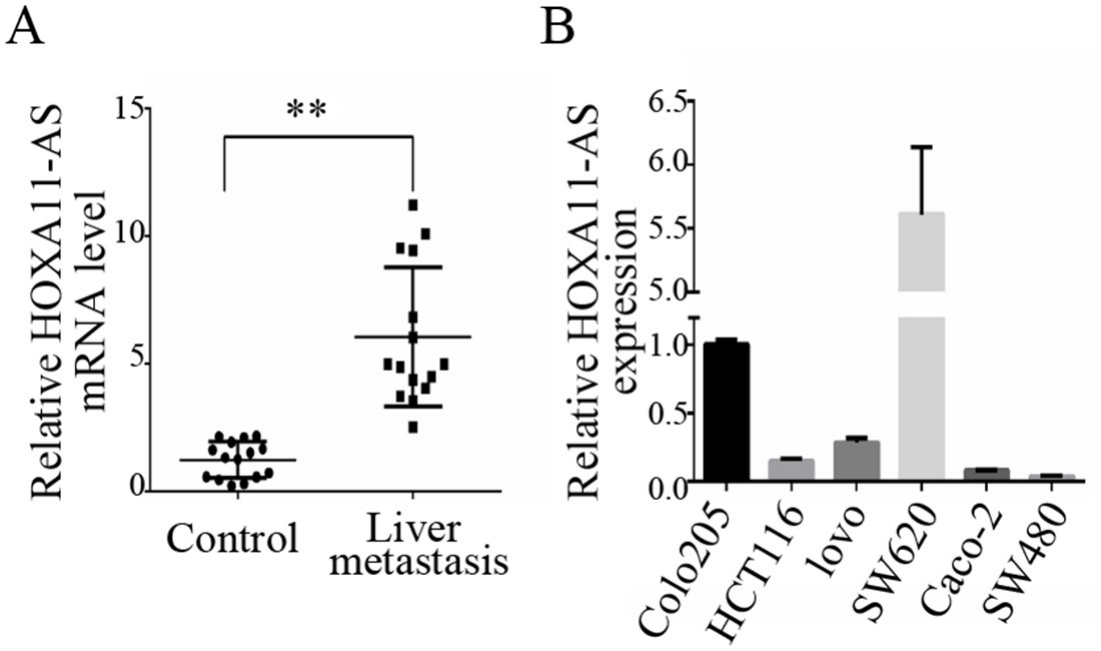
HOMEOBOX A11 antisense RNA (HOXA11-AS) expression level in colorectal cancer (CRC) patients with liver metastasis **(A)** The expression of HOXA11-AS was significantly increased in CRC tissues with liver metastasis compared to those without. **(B)** HOXA11-AS expression in six CRC cell lines. **: p < 0.01.

Quantitative real-time polymerase chain reaction (qRT-PCR) revealed a significant increase (p<0.0001) in HOXA11-AS expression in 15 CRC patients with liver metastasis compared to those without metastasis (Figure 1A). HOXA11-AS expression was detected in six CRC cell lines (Figure 1B); the expression levels in highly invasive cell lines (Colo205, HCT116, Lovo, and SW620) were higher than those in minimally invasive cell lines (Caco-2 and SW480). Those cell lines with the highest (SW620) and lowest (SW480) HOXA11-AS expression levels were selected for subsequent analysis.

### HOXA11-AS promotes the migration and invasion of CRC cells

**Figure 2:**
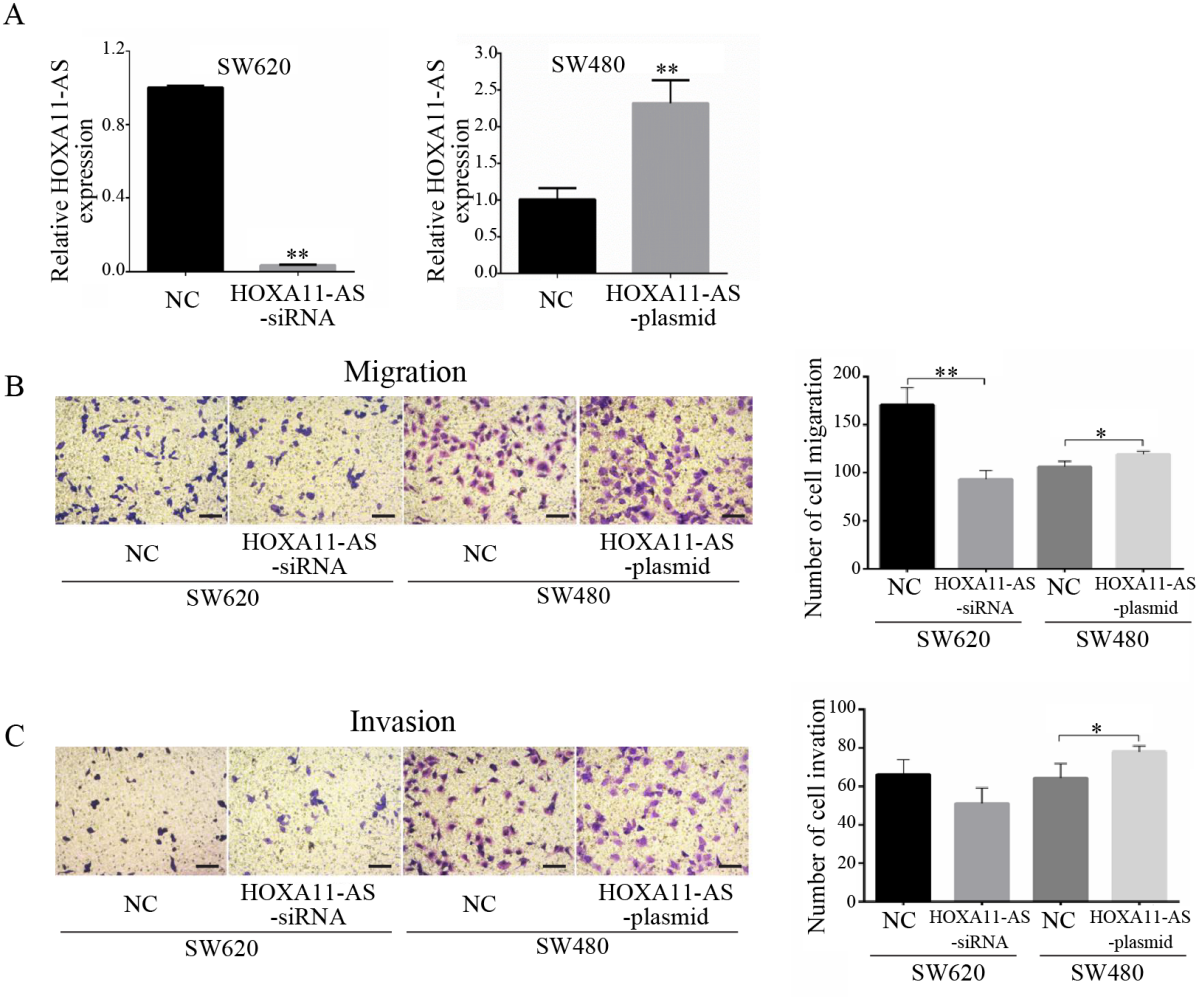
HOXA11-AS promotes the invasion and metastasis of CRC cells **(A)** HOXA11-AS siRNA transfection into SW620 cells significantly reduced the expression of HOXA11-AS, while HOXA11-AS-plasmid significantly increased the expression of HOXA11-AS in SW480 cells. **(B)** HOXA11-AS siRNA transfection into SW620 cells significantly reduced cell migration, while HOXA11-AS-plasmid significantly increased cell migration. **(C)** HOXA11-AS siRNA transfection into SW620 cells dramatically reduced cell invasion, while HOXA11-AS-plasmid significantly increased cell invasion. *: p < 0.05, **: p < 0.01. Scale bar: 50um.

To determine the function of HOXA11-AS in CRC metastasis, the transfection of an siRNA targeting HOXA11-AS and the overexpression of HOXA11-AS in SW480 cells were validated by qRT-PCR analysis. HOXA11-AS-siRNA caused a significant decrease (p<0.0001) in HOXA11-AS expression in SW620 cells, while transfection with the HOXA11-AS-plasmid resulted in a significant increase (p=0.0028) in SW480 cells (Figure 2A). The transfection of HOXA11-AS siRNA into SW620 cells significantly reduced cell migration (p=0.0026), and HOXA11-AS overexpression in SW480 cells increased cell migration significantly (p=0.0324) (Figure 2B). Similarly, the transfection of HOXA11-AS siRNA into SW620 cells resulted in a dramatic reduction in cell invasion, and the overexpression of HOXA11-AS in SW480 cells caused a significant increase in cell invasion (p=0.0438) (Figure 2C).

### HOXA11-AS acts as a molecular sponge for miR-125a-5p

**Figure 3:**
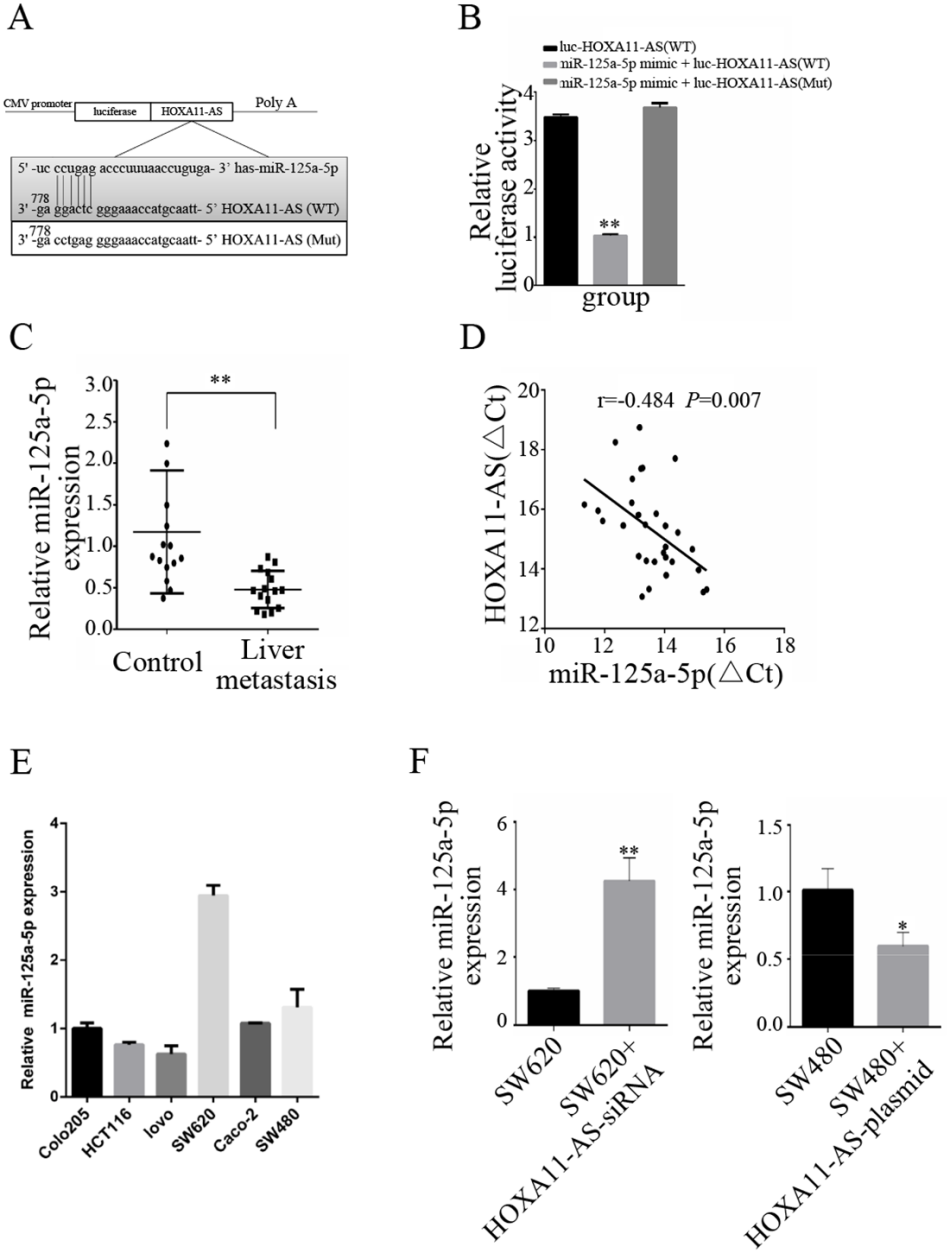
HOXA11-AS acts as a molecular sponge for miR-125a-5p **(A)** The putative miR-125a-5p-binding sequence of HOXA11-AS. A mutation was generated in the HOXA11-AS sequence in the complementary site for the seed region of miR-125a-5p. **(B)** 293T cells were transfected with wild-type HOXA11-AS (WT), miR-125a-5p mimic + HOXA11-AS (WT), or miR-125a-5p mimic + mutated HOXA11-AS (Mut). Luciferase activity was measured 48 h after transfection using a dual-luciferase reporter gene assay. **(C)** Expression of miR-125a-5p in 15 pairs of CRC patient samples with liver metastasis and CRC patients without metastasis. **(D)** The correlation between HOXA11-AS and miR-125a-5p was measured in 15 pairs of CRC patient samples with liver metastasis and CRC patients without metastasis using Pearson’s correlation analysis. r =−0.484; p = 0.007; **(E)** qRT-PCR analysis of miR-125a-5p expression in six CRC cell lines. **(F)** SW620 cells were transfected with NC or HOXA11-AS-siRNA; SW480 cells were transfected with NC or HOXA11-AS-plasmid respectively. qRT-PCR was used to detect miR-125a-5p expression. *: p < 0.05, **: p < 0.01.

Based on our previous ceRNA analysis, we performed a bioinformatics analysis using Starbase 2.0 (http://starbase.sysu.edu.cn) and found that HOXA11-AS and miR-125a-5p contain complementary base pairs (Figure 3A), indicating that HOXA11-AS may act as a sponge in the deregulation of miR-125a-5p. To confirm this observation, we constructed luciferase reporters carrying HOXA11-AS that contained wild-type (WT) or mutant (Mut) miR-125a-5p binding sites for investigation. Luciferase reporters were transfected together with miR-125a-5p mimics in CRC cells. The predicted miR-125a-5p bound to the HOXA11-AS fragment containing the miRNA target sites. This resulted in a 71% decrease in luciferase activity in 293T cells relative to the empty vector control (Figure 3B). These results indicate that miR-125a-5p binds directly to HOXA11-AS. qRT-PCR revealed a significant decrease (p=0.0016) in miR-125a-5p expression in 15 CRC patients with liver metastasis compared to those without metastasis (Figure 3C). Moreover, the expression of miR-125a-5p was significantly negatively correlated (p=0.007) with HOXA11-AS expression (Figure 3D). miR-125a-5p expression was detected in six CRC cell lines (Figure 3E).

The effect of HOXA11-AS on the endogenous expression of miR-125a-5p was further examined. qRT-PCR analysis revealed that the overexpression of HOXA11-AS substantially decreased the expression of miR-125a-5p in SW480 cells, and that the knockdown of HOXA11-AS increased miR-125a-5p expression in SW620 cells (Figure 3F).

### HOXA11-AS promotes CRC metastasis by binding competitively to miR-125a-5p

**Figure 4:**
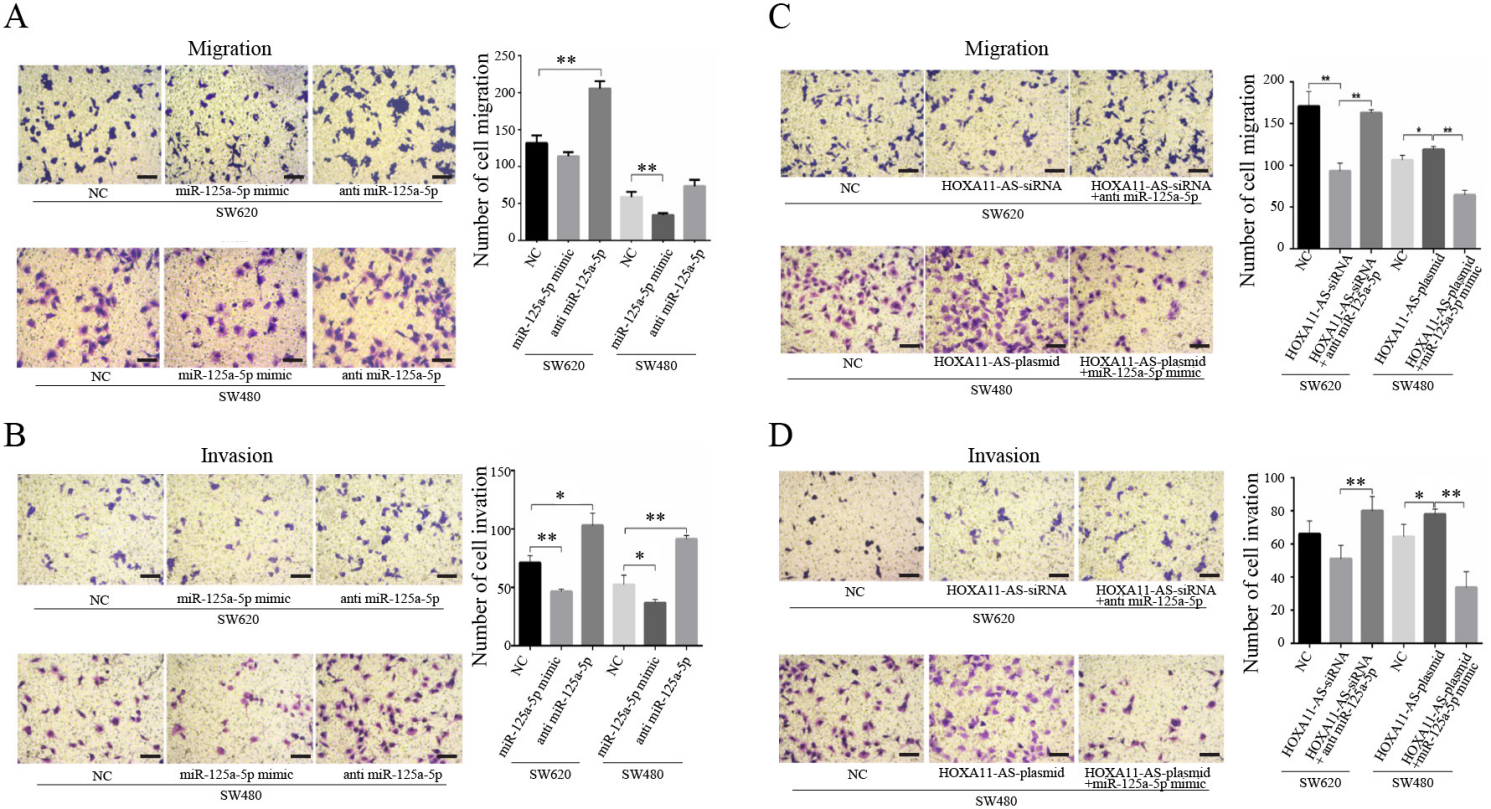
HOXA11-AS promotes CRC metastasis by binding competitively to miR-125a-5p Migration (**4A** and **4C**) and invasion (**4B** and **4D**) assays revealed the relationship between HOXA11-AS and miR-125a-5p in regulating CRC metastasis. *: p < 0.05, **: p < 0.01. Scale bar: 50um.

Migration and invasion assays were performed to verify the functional roles of HOXA11-AS and miR-125a-5p in the promotion of CRC metastasis. First, the miR-125a-5p mimic or anti-miR-125a-5p was applied to SW480 and SW620 cells for migration and invasion assays, respectively. Cells in the lower chamber were altered when treated with the miR-125a-5p mimic or anti-miR-125a-5p compared to untreated cells in both the migration and invasion assays (Figure 4A and 4B). To further confirm the relationship between HOXA11-AS and miR-125a-5p in the regulation of CRC metastasis, HOXA11-AS-siRNA and HOXA11-AS-siRNA plus anti-miR-125a-5p were examined in SW620 cells. The migration and invasion of HOXA11-AS-plasmid and HOXA11-AS-plasmid plus the miR-125a-5p mimic were also examined in SW480 cells. HOXA11-AS promoted CRC metastasis by binding competitively to miR-125a-5p, as confirmed in both the migration (Figure 4C) and invasion (Figure 4D) assays.

### HOXA11-AS modulates the expression of the endogenous miR-125a-5p target PADI2

**Figure 5:**
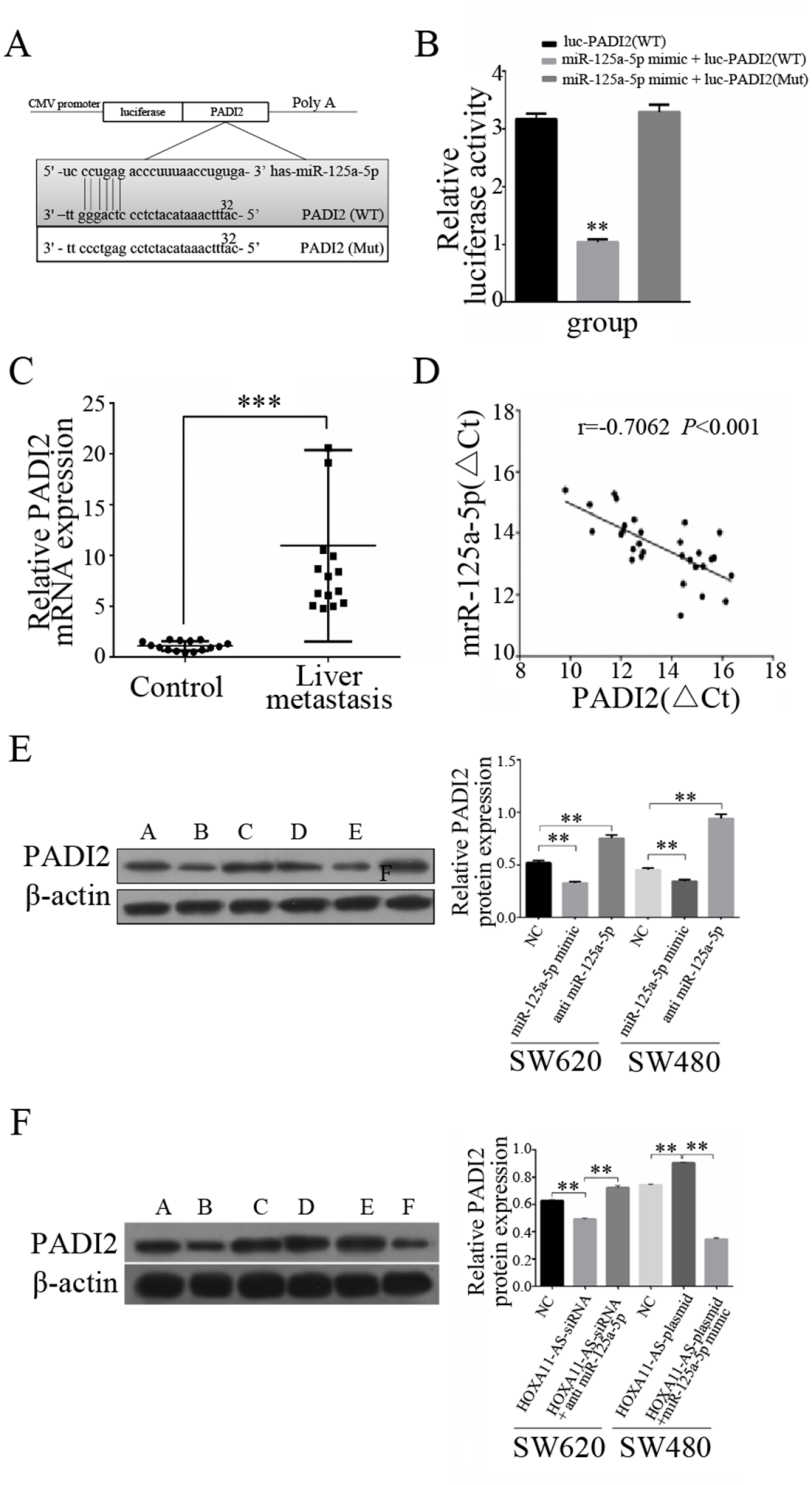
HOXA11-AS modulates the expression of the endogenous miR-125a-5p target PADI2 **(A)** The putative miR-125a-5p-binding sequence of PADI2. A mutation was generated in the PADI2 sequence in the complementary site for the seed region of miR-125a-5p. **(B)** 293T cells were transfected with PADI2 (WT), miR-125a-5p mimic + PADI2 (WT), or miR-125a-5p mimic + PADI2 (Mut). Luciferase activity was measured 48 h after transfection using a dual-luciferase reporter gene assay. **(C)** The expression of PADI2 was significantly increased in CRC tissues with liver metastasis compared to those without. **(D)** The correlation between PADI2 and miR-125a-5p was measured in 15 pairs of CRC patient samples with liver metastasis and CRC patients without metastasis using Pearson’s correlation analysis. r =−0.7062; p <0.001; **(E)** PADI2 protein expression was examined by Western blotting following treatment with an miR-125a-5p mimic or anti-miR-125a-5p, as well as untreated cells (negative control, NC) in both SW620 and SW480 cells. **(F)** PADI2 protein expression was examined by Western blotting following treatment with HOXA11-AS-siRNA, HOXA11-AS-siRNA + anti-miR-125a-5p, and the NC in SW620 cells, and HOXA11-AS-plasmid, HOXA11-AS-plasmid + miR-125a-5p mimic, and the NC in SW480 cells. **: p < 0.01. ***: p <0.001.

In our previous ceRNA analysis, we found that PADI2 and miR-125a-5p contain complementary base pairs (Figure 5A), indicating that PADI2 may act as a sponge in the deregulation of miR-125a-5p. To confirm this, we constructed luciferase reporters carrying PADI2 that contained WT or Mut miR-125a-5p binding sites for investigation. We mutated the candidate binding site and found that the WT 3'-untranslated region (UTR) of PADI2 exhibited a lower translation level in the presence of miR-125a-5p, whereas the mutated 3'-UTR did not show a significant response to miR-125a-5p (Figure 5B).

These results indicate that miR-125a-5p is a PADI2-targeting miRNA. Moreover, the inverse correlation between miR-125a-5p and PADI2 mRNA expression was verified by a linear regression analysis in 15 CRC patients with liver metastasis compared to those without metastasis (Figure 5D). We examined the expression levels of PADI2 in CRC patients and determined that PADI2 expression was higher in patients with liver metastasis compared to those without metastasis (Figure 5C).

Western blot analysis was used to verify the functions of HOXA11-AS and miR-125a-5p in PADI2 regulation. Both the miR-125a-5p mimic and anti-miR-125a-5p were proven to significantly alter PADI2 protein expression in SW620 and SW480 cells (Figure 5E). Moreover, the examination of HOXA11-AS-siRNA and HOXA11-AS-siRNA plus anti-miR-125a-5p in SW620 cells, as well as HOXA11-AS-plasmid and HOXA11-AS-plasmid plus the miR-125a-5p mimic in SW480 cells, revealed that HOXA11-AS modulates the expression of the endogenous miR-125a-5p target PADI2 (Figure 5F).

### PADI2 promotes the metastatic ability of CRC

**Figure 6:**
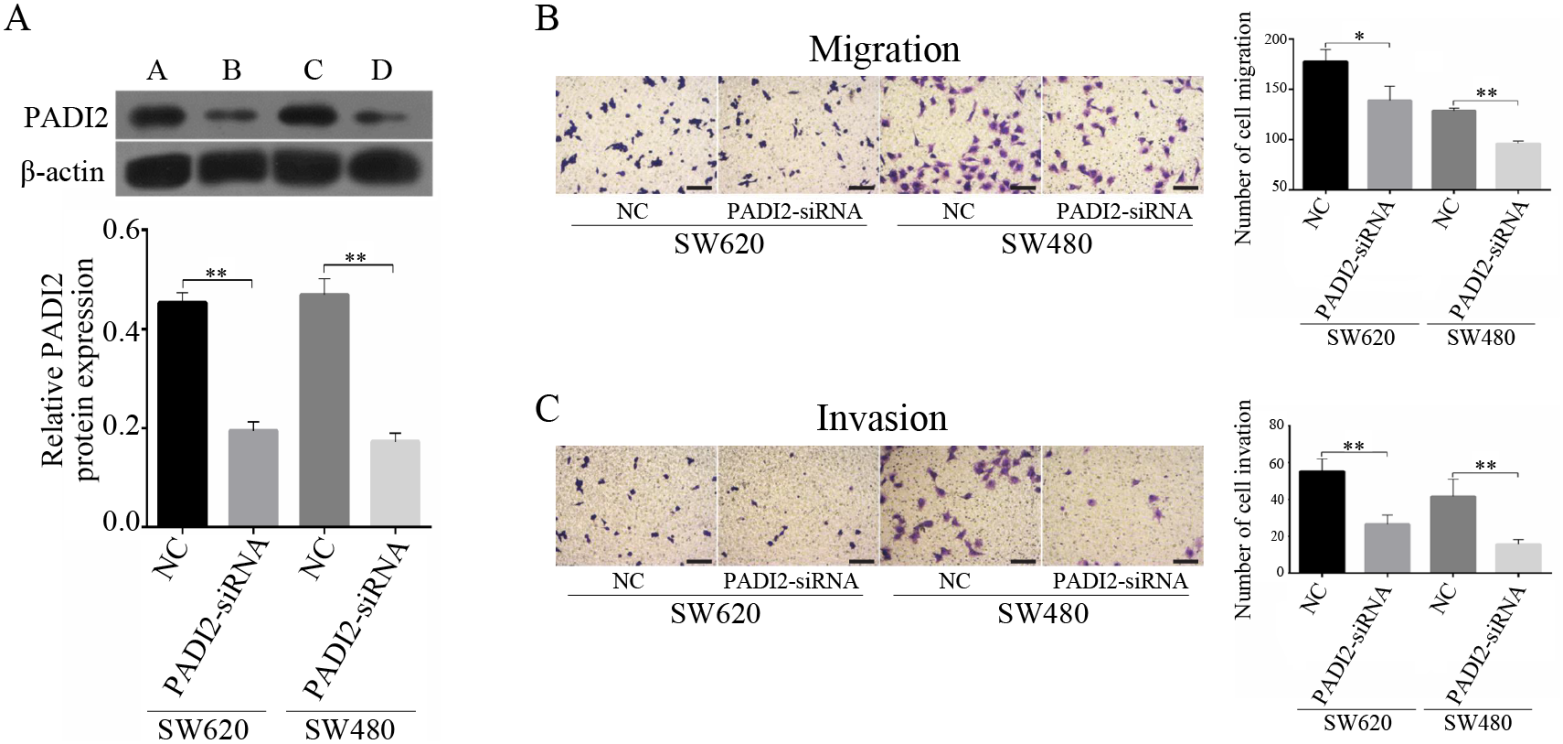
PADI2 promotes the metastatic ability of CRC cells **(A)** PADI2 siRNA transfection significantly reduced the expression of PADI2 in both SW620 and SW480 cells. **(B)** PADI2 siRNA transfection significantly reduced cell migration in both SW620 and SW480 cells. **(C)** PADI2 siRNA transfection significantly reduced cell invasion in both SW620 and SW480 cells. *: p < 0.05, **: p < 0.01. Scale bar: 50um.

To further determine the ability of PADI2 to promote CRC metastasis, the effects of PADI2 siRNA transfection were verified in both SW620 (p<0.0001) and SW480 (p=0.0002) cells by Western blot analysis (Figure 6A). PADI2 siRNA transfection reduced cell migration significantly in both SW620 (p=0.0233) and SW480 (p=0.0002) cells (Figure 6B). Similarly, PADI2 siRNA transfection significantly reduced cell invasion in both SW620 (p=0.0039) and SW480 (p=0.0095) cells (Figure 6C).

## DISCUSSION

HOXA11-AS expression is related to many cancers. We previously reported that the expression of HOXA11-AS was significantly up-regulated in tissues of CRC patients with liver metastasis [30]. In this study, qRT-PCR analysis revealed that the expression of HOXA11-AS was significantly increased in CRC samples with liver metastasis compared to those without metastasis. Moreover, the knockdown and overexpression of HOXA11-AS inhibited and promoted the migration and invasion, respectively, of both selected colon cancer cells.

The presence of ceRNAs, which are involved in an intricate interplay between communicating with and co-regulating each other by binding competitively to shared miRNAs or messenger RNAs/proteins, may explain the mechanism of ceRNA regulation in cancer [8]. Increasing evidence suggests that the ceRNA activity of lncRNAs plays a critical role [31]. A growing number of lncRNAs act as “sponges” to bind to specific miRNAs to regulate epithelial ovarian cancer, pancreatic adenocarcinoma, gastric cancer, clear cell kidney carcinoma, and gallbladder cancer [32–36].

We previously detected the overexpression of HOXA11-AS and PADI2 in CRC tissues with liver metastasis (compared to CRC tissues without metastasis) [30]. Furthermore, we determined that HOXA11-AS is related to PADI2 expression in the progression of liver metastasis in CRC, and that both may be directly related to miR-125a-5p based on coding-non-coding and ceRNA analyses [30]. Based on these results, we developed a model in which HOXA11-AS binds competitively to miR-125a-5p to overexpress PADI2, and overexpressed PADI2 subsequently promotes CRC metastasis.

In the present study, significant alterations in HOXA11-AS, miR-125a-5p, and PADI2 expression were identified in a cohort of 15 patients with CRC and liver metastasis and 15 patients with CRC without metastasis. Moreover, the expression of miR-125a-5p was significantly negatively correlated with both HOXA11-AS and PADI2, indicating their clinical importance in CRC liver metastasis. Thereafter, two classic CRC cell lines with relatively high or low expression levels of HOXA11-AS were chosen for additional *in vitro* studies. Overexpression or knockdown studies of HOXA11-AS or PADI2, as well as gain-/loss-of-function studies of miR-125a-5p, revealed a positive correlation between HOXA11-AS and PADI2 and a negative correlation with miR-125a-5p, confirming their involvement in the regulation of liver metastasis in CRC cell lines.

Using dual-luciferase reporter gene assays, we identified HOXA11-AS and PADI2 as endogenous sponges for miR-125a-5p. The competitive relationship between HOXA11-AS and PADI2 represents a key step in the previously unknown mechanism of the HOXA11-AS-induced promotion of liver metastasis in CRC. In a future study, we will employ RNA pull-down assays to verify the direct binding between HOXA11-AS and miR-125a-5p.

Collectively, our study supports a role for HOXA11-AS in the promotion of liver metastasis by functioning as a ceRNA sponge for miR-125a-5p to promote PADI2 expression. Our findings indicate a novel HOXA11-AS/miR-125a-5p/PADI2 regulatory network in CRC liver metastasis. Our findings also show that HOXA11-AS is an important molecular marker in predicting liver metastasis and a potential target for CRC therapy.

## MATERIALS AND METHODS

### Human tissue samples and cell lines

Samples were collected from patients admitted to the Department of Colorectal Surgery, First Affiliated Hospital, Zhejiang University, Hangzhou, China, between April 2016 and September 2016. In total, 30 primary CRC samples were obtained from 15 patients with CRC and liver metastasis and 15 patients with CRC without metastasis. None of the patients had received neoadjuvant therapy. The colorectal samples were pathologically confirmed postoperatively as colorectal adenocarcinoma, and the liver samples were confirmed as metastatic adenocarcinoma by operation or biopsy. Samples were taken within 10 min of tumor excision, immediately immersed in RNAlater™ Stabilization Solution (Thermo Fisher Scientific, Waltham, MA, USA) and stored at -80°C until use. Written informed consent was obtained from all patients. This study was approved (2016-40) by the Ethics Committee of First Affiliated Hospital, College of Medicine, Zhejiang University.

Human Colo205, HCT116, Lovo, SW620, Caco-2, and SW480 CRC cells were obtained from Tongpai Biological Technology (Shanghai, China). HCT116, SW620, and SW480 cells were cultivated in RPMI-1640 medium (Gibco, Carlsbad, CA, USA). Colo205 cells were cultivated in Dulbecco’s Modified Eagle’s Medium (Gibco). Lovo cells were cultivated in F12K medium (Gibco). All cell lines described above were supplemented with 10% fetal bovine serum (FBS; HyClone Laboratories, Logan, UT, USA). Caco-2 cells were cultivated in Minimal Essential Media (Gibco) supplemented with 20% FBS (HyClone). All cells were maintained in a 37°C incubator with a humidified atmosphere containing 5% CO_2_.

### RNA extraction and qRT-PCR

Total RNA was isolated using TRIzol reagent (Invitrogen, Carlsbad, CA, USA) according to the manufacturer’s instructions. RNA samples were reverse-transcribed into cDNA with different primers using a Prime Script Kit (Takara Bio Inc., Otsu, Japan). qRT-PCR was used to analyze the mRNAs and lncRNAs, whose expression was normalized to that of β-actin. A SYBR Premix Ex Taq™ Kit (Takara Bio Inc.) was used for the qRT-PCR assay. All qRT-PCRs were performed in triplicate. The relative level of gene expression is presented as ΔCt=Ct_gene_ – Ct_reference_. The fold change in gene expression was calculated using the 2^−ΔΔCt^ formula. All primer sequences are shown in Table 1.

**Table 1:** The sequence of the prime

Gene	Forward primer	Reverse primer
HOXA11-AS	GAGTGTTGGCCTGTCCTCAA	TTGTGCCCAGTTGCCTGTAT
miR125a-5p	TGCGGCTCCCTGAGACCCTTTAA	
PADI2	GGGATGAGCAGCAAGCGAAT	TGAGGATGTCACGGTTCCAG
U6	CTCGCTTCGGCAGCACA	AACGCTTCACGAATTTGCGT
β-actin	TGAGGATGTCACGGTTCCAG	GTCACCTTCACCGTTCCAGT

### Construct generation and transient transfection

To study the effects of HOXA11-AS, miR-125a-5p, and PADI2 on cell activity, miR-125a-5p mimics, miR-125a-5p inhibitors, HOXA11-AS-siRNAs (for the knockdown of HOXA11-AS expression), and PADI2-siRNAs were obtained from Gene Pharma (Shanghai, China). The lncRNA-HOXA11-AS cDNA plasmid was constructed by introducing the cDNA sequence of HOXA11 into the pEX3 expression vector (Gene Pharma).

The human CRC cell lines SW620 and SW480 (2 × 10^5^ cells) were transfected with miRNA mimics, miRNA inhibitors, PADI2-siRNAs, or HOXA11-AS-siRNAs at a final concentration of 25 nmol/L using Lipofectamine 2000 Reagent (Life Technologies, Carlsbad, CA, USA). Cells were transfected with pcDNA- lncRNA-HOXA11-AS constructs at a final concentration of 1 μg/μL according the manufacturer’s protocol. Forty-eight hours after transfection, total RNA from the harvested cells was isolated with TRIzol reagent (Invitrogen). The empty pEX3 vector and scrambled sequences of the miRNA mimics, miRNA inhibitors, or siRNAs were used as negative controls (NCs).

### Western blot analysis

Cell lysates were homogenized in Cell Lysis Solution (Sigma-Aldrich, St. Louis, MO, USA) and centrifuged for 5 min at 4°C. Samples were subjected to sodium dodecyl sulfate-polyacrylamide gel electrophoresis for 3 h and then transferred to polyvinylidene fluoride membranes (Amersham Biosciences, Piscataway, NJ, USA) for an additional 2 h. Antibodies against human PADI2 (Proteintech, Rosemont, IL, USA; 2,000-fold dilution) were used for Western blot analysis. After incubation with specific antibodies against β-actin (Sigma-Aldrich) at 4°C overnight, the membranes were washed with 1% Tris-buffered saline containing Tween 20 in triplicate, incubated with the appropriate secondary antibodies for 1 h and detected by chemiluminescence.

### Dual-luciferase reporter gene assays

Two luciferase reporters containing WT HOXA11-AS (luc-HOXA11-AS-WT) or Mut HOXA11-AS were generated to analyze the interaction between HOXA11-AS and miR-125a-5p. Mut HOXA11-AS contained a mutation site (luc-HOXA11-AS-MU) that abolishes targeting by miR-125a-5p. 293T cells were seeded onto 24-well plates and co-transfected with a luciferase reporter and the miR-125a-5p mimic or NC. Cells were lysed according to the instructions provided with the Dual-Luciferase Reporter Assay System (Promega, Madison, WI, USA). Luciferase activity was measured 48 h after transfection according to the manufacturer’s instructions on a Panomics Luminometer (Affymetrix, Santa Clara, CA, USA). Luciferase activity was normalized to that of *Renilla* luciferase.

### Matrigel invasion and transwell migration assays

Cells were trypsinized and seeded at a density of 50,000 cells/well on both Matrigel-coated (BD Biosciences, San Jose, CA, USA) and uncoated (Corning, Tewksbury, MA, USA) transwell filters in a 24-well plate. Both groups of cells were allowed to invade for 12 h toward 10% FBS in the lower chambers. Three-Step Stain (Richard-Allan Scientific, San Diego, CA, USA) was applied when the cells invaded or migrated through the Matrigel-coated or uncoated filters, respectively. Each filter was counted in its entirety under the rule of four 10× fields, while invasion or migration was quantified as the fold change relative to the control.

### Statistical analysis

All statistical analyses were performed using the SPSS 16.0 software package (SPSS Inc., Chicago, IL, USA). Significant differences between two groups were estimated by Student’s *t*-test, the Wilcoxon signed-rank test, or Pearson’s chi-square test as appropriate. Pearson’s correlation analysis was used to estimate the relationship between the expression of HOXA11-AS and miR-125a-5p, as well as miR-125a-5p and PADI2; p<0.05 was considered statistically significant.

## Abbreviations

(CRC): Colorectal cancer
(CeRNAs): competing endogenous RNAs
(HOXA11-AS): HOXA11 antisense RNA
(lncRNA): long noncoding RNA
(miRNA): MicroRNA
(PADI2): peptidyl arginine deiminase 2
qRT-PCR: quantitative real-time polymerase chain reaction
